# Cognitive Impact of Cerebellar Non-invasive Stimulation in a Patient With Schizophrenia

**DOI:** 10.3389/fpsyt.2020.00174

**Published:** 2020-03-17

**Authors:** Charles Laidi, Carole Levenes, Alex Suarez-Perez, Caroline Février, Florence Durand, Noomane Bouaziz, Dominique Januel

**Affiliations:** ^1^Pôle de Psychiatrie, Assistance Publique–Hôpitaux de Paris (AP-HP), Faculté de Médecine de Créteil, DMU IMPACT, Hôpitaux Universitaires Mondor, Créteil, France; ^2^Institut National de la Santé et de la Recherche Médicale (INSERM), U955, Institut Mondor de Recherche Biomédicale, Psychiatrie Translationnelle, Créteil, France; ^3^UNIACT, Psychiatry Team, Neurospin Neuroimaging Platform, CEA Saclay, Gif-sur-Yvette, France; ^4^Fondation Fondamental, Créteil, France; ^5^Integrative Neuroscience and Cognition Center (INCC UMR8002), Centre National de la Recherche Scientifique (CNRS), Institute for Neuroscience and Cognition, University of Paris, Paris, France; ^6^Hôpital de Ville Evrard, Unité de Recherche Clinique, Neuilly-sur-Marne, France

**Keywords:** schizophrenia, tDCS—transcranial direct current stimulation, cerebellum, eyeblink conditioning, cognition

## Abstract

Cerebellum plays a role in the regulation of cognitive processes. Cerebellar alterations could explain cognitive impairments in schizophrenia. We describe the case of a 50 years old patient with schizophrenia whom underwent cerebellar transcranial direct current stimulation (tDCS). In order to study the effect of cerebellar stimulation on cognitive functions, the patient underwent a neuropsychological assessment and an eyeblink conditioning (EBC) protocol. Although the effect of brain stimulation cannot be only assessed in a single-case study, our results suggest that cerebellar stimulation may have an effect on a broad range of cognitive functions typically impaired in patients with schizophrenia, including verbal episodic, short term, and working memory. In addition to neuropsychological tests, we evaluated the cerebellar function by performing EBC before and after tDCS. Our data suggest that tDCS can improve EBC. Further clinical trials are required for better understanding of how cerebellar stimulation can modulate cognitive processes in patients with schizophrenia and healthy controls.

## Background

The cerebellum is involved in a broad range of cognitive functions, including working memory, emotion processing, and social cognition (1). In humans, the cerebellum represents 10% of the brain volume but contains more than 50% of its neurons (1). The posterior lobe of the cerebellum is involved in cognition and connected to associative regions such as the prefrontal cortex, whereas the anterior cerebellum is known to modulate sensory-motor cortical activity (2). As alterations of the sensory-motor parts of the cerebellum lead to motor dysmetria, abnormalities in the posterior cerebellum may have implications for cognitive dysmetria. Andreasen et al. (3) have proposed that abnormalities in the posterior cerebellum may explain some symptoms of schizophrenia.

Schizophrenia is a severe mental disorder characterized by the association of positive, negative, and cognitive symptoms. Cognitive symptoms, that often precedes the illness, have an impact on the quality of life and on the functioning of the patients (4). Deficits in working memory, attention, processing speed, visual, and verbal procedural learning have been documented in schizophrenia (5). However, pharmacological interventions only have a very limited impact on cognitive deficits in schizophrenia. Although non-pharmacological interventions, such as cognitive remediation can improve cognitive deficits in patients, there is a clear need for new interventions to target cognitive symptoms in schizophrenia.

Neuropsychological tests are commonly used to assess cognitive functions in patients with schizophrenia. These tests require full cooperation of participants, which can be difficult in a population of patients with schizophrenia suffering from motivational deficits (6).

Eyeblink conditioning (EBC) does not rely on motivation of the subject. It is based on a simple reflex pathway and measures associative learning (7). EBC is a form of classical conditioning that consists of pairing a stimulus (conditioned stimulus—CS, auditory in our study) with an unconditioned stimulus (US, airpuff in our study) that induces an eyeblink reflex. In delay-type EBC, a tone CS precedes and co-terminates with a corneal airpuff US that elicits an unconditioned response (UR). Over repeated pairings, the CS induces a conditioned response (CR) that precedes and reduces the US. McCormick et al. first showed that electrolytic lesions of the ipsilateral cerebellum completely prevented the acquisition and retrieval of the delay EBC (8, 9). The abundant literature based on lesion, reversible inactivation, genetic manipulation, electrical stimulation, optogenetics, electrophysiology, and brain-imaging studies show that the cerebellum is necessary and sufficient for acquisition, expression, and extinction of EBC provided that the interval between CS and US stays in the range of 1 s [see review in (10, 11)]. In accordance with animal research, EBC is a relevant method to investigate cerebellar dysfunction in schizophrenia disorders (12).

Non-invasive brain stimulation techniques are commonly used in healthy adults and patients with neuropsychiatric disorders to investigate brain mechanisms or to enhance cognitive, behavioral, social, and emotion processes (13). Transcranial direct current stimulation (tDCS) is a form of neuromodulation delivering a low direct constant current over two electrodes placed on the scalp. Applied to the cerebellum, tDCS can deliver an electric field reaching the cerebellum at a strength within the range of values for modulating activity in the cerebellar neurons (14).

In healthy subjects, two studies (15, 16) reported an effect of cerebellar tDCS on EBC. Interestingly, van der Vliet et al. (16) reported an interaction between the effect of cerebellar tDCS on EBC and the BDNF Val66Met polymorphism, previously involved in cognitive deficits in schizophrenia (17).

We describe the case of a 50 years old patient with schizophrenia whom underwent posterior cerebellar transcranial direct current stimulation (tDCS). We report neuropsychological testing and monitoring of cerebellar function with EBC before and after 1 week of stimulation in the posterior cerebellum.

## Case Presentation

The patient was a 50 years old man suffering from schizophrenia. During the stimulation period, the patient was stabilized under a treatment of intramuscular haloperidol (150 mg every 4 weeks) and Zopiclone (7.5 mg/j). There was no change in the patient medication during the assessment and stimulation protocol. The patient was married with two children and was discontinuously working in the construction sector. He was mostly complaining from auditory hallucinations: the patient reported that he was hearing at least once a day a male voice that was giving him orders.

Written consent was obtained from the participant prior to the study.

The patient underwent 5 days of tDCS stimulation. The post-tDCS EBC session was performed 5 days after tDCS; 7 days separated the two EBC sessions. The cerebellum was stimulated using a NeuroConn DC Stimulator (NeuroConn GmbH) with two 5 × 7 cm conductive-rubber electrodes placed over the cerebellum, 1 cm below the inion (anode) and on the right arm (cathode). Stimulation was administered during two sessions of 25 min (separated by 1 h), including 5 s of ramp-up and 5 s of ramp-down, with an intensity of 2 mA (for a total of 10 stimulation sessions). The patient was stimulated during 5 consecutive days for a total of 10 sessions. We chose this stimulation protocol based on a previous modeling study (18) and on the work of Ferruci et al. (14).

**Figure 1:**
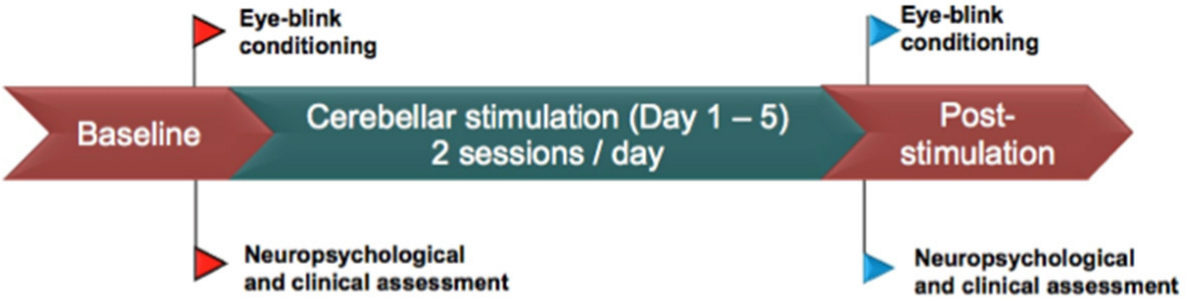
Stimulation protocol and clinical assessment.

Clinical assessment included the Positive And Negative Symptom Scale (PANSS) (19) and the Auditory Hallucination Rating Scale (AHRS), before and after stimulation. Neuropsychological assessment explored key cognitive functions typically impaired in patients with schizophrenia: episodic memory, executive, and attentional functions. We selected neuropsychological tests with no test/retest effect in order to compare neuropsychological outcomes before and after stimulation (20, 21). The patient underwent a long term episodic memory test (French version of Free and cued recall−16 items, Grober & Buschke, measuring anterograde episodic verbal memory using two different verbal material) (21), two subtests of the Wechsler Adult Intelligence Scale (WAIS-IV) (digit span and spatial memory), the stroop test (Golden version) (22) and the D2 Test of attention (Brickenkamp) (20). Neuropsychological assessment was repeated 2 days before and 2 days after the stimulation protocol (Figure 1) by a trained neuropsychologist that was not involved in the conception of the study nor in the brain stimulation.

The conditioning of the eyeblink reflex (EBC) was performed with a portable human eyeblink conditioning system (San Diego Instruments). The system included an infrared (IR) reflective sensor glued together with small 1.5 mm air-delivering tubing positioned just beneath the superior eyelid of the subject. The EBC device, comprising a portable airpuff and headset sound-delivering unit, controlled the timing, and intensity of both the airpuff and the sound (pure tone). It also converted the analog IR-reflection signal to numeric and sent it to a personal computer. The IR-reflection signal was collected online with the San Diego Instruments Labview software and then analyzed offline with a custom-made routine under Python (Python Software Foundation). During the overall experiment a continuous background white noise was delivered to the subject through the headset in order to provide constant ambient noise. The conditioning stimulus (CS) consisted of a 400 ms−1.2 kHz pure tone. We set the intensity of the CS such as it did not trigger any startle reflex nor any detectable reaction from the subject, thereby reducing the occurrence of alpha responses. The unconditioning stimulus (US) consisted of a 50 ms airpuff whose intensity (air pressure at the tip of the tube) was set to trigger painless eyeblink in 100% of the trials. Initially, the participant was exposed to five CS alone and to five US alone stimuli to establish appropriate responses to the tone and the airpuff as well as to measure the UR prior to conditioning. We also ensured that the US did not induce any startle reaction from the participant. A conditioning trial lasted 1 s and consisted of (successively): an initial 200 ms baseline period, a 400 ms CS that co-terminated with a 50 ms US, and a final 400 ms period during which the eyeblink was recorded. An EBC block consisted of 9 successive paired presentations of CS-US and a last trial with the CS alone. An EBC session consisted of 5 successive blocks separated by an inter-trial interval randomly ranging from 2 to 12 s. The participant was passively watching a silent movie during the task.

EBC sessions: The patient underwent two EBC sessions on the same days as the clinical and neuropsychological assessments (Figure 1). The first one (pre-tDCS) was made two days before the first tDCS session, and the second one (post-tDCS) was made 2 days after the last tDCS session. Thus, 7 days separated the pre- and the post-tDCS EBC sessions.

The EBC signal was low-pass filtered using a 4th order Butterworth filter with a 10 Hz cutoff frequency and was offset-subtracted by deducing to the trace the averaged baseline. To estimate the percentage of CRs, we discarded trials for which a spontaneous blink occurred during the baseline. CRs were detected in a time window between 330 and 400 ms after the CS onset with a threshold of five times the standard deviation above the baseline. We visualized each trace separately afterwards to verify that the detection of CRs was correct.

Clinical and neuropsychological characteristics before and after cerebellar stimulation are reported in Table 1. The patient did not report any side effects, except from a slight itching in the beginning of the first session of the second day of stimulation. A careful inspection of the scalp did not evidence any cutaneous lesion. The patient did not report any headache after brain stimulation. Clinical symptoms remained stable during the stimulation protocol. Notably there was no changes in the PANSS score and the Auditory Hallucination Verbal Scale.

**Table 1:** Clinical and neuropsychological characteristics before and after cerebellar stimulation.

	**Before stimulation**	**After stimulation**
**CLINICAL ASSESSMENT**		
PANSS score:		
- Total - Positive - Negative - General psychopathology	57 22 15 20	58 24 14 20
AHRS	22	23
**NEUROPSYCHOLOGICAL ASSESSMENT**		
**Free and cued recall 16 items:**		
- Recall 1		
- Free recall (pc) - Total recall (pc)	5 (1–2) 8 (1)	7 (11–12) 11 (1–5)
- Recall 2		
- Free recall (pc) - Total recall (pc)	5 (<0.1) 10 (<1)	7 (3–4) 13 (5)
- Recall 3		
- Free recall (pc) - Total recall (pc)	7 (<1) 9 (<1)	8 (1–2) 12 (1)
- Delayed recall		
- Free recall (pc) - Total recall (pc)	7 (<1) 9 (<1)	11 (19–20) 11 (1)
**WAIS-IV:**		
- Digit span		
- Direct order - Indirect order - Increasing order - Total	5 4 6 15	6 5 6 17
- Spatial memory		
- Direct order - Indirect order - Total	6 6 12	9 6 15
Stroop-test golden version^*^:		
- Reading (pc) - Denomination (pc) - Interference (pc)	74 (4–5) 62 (12) 30 (5–8)	82 (8–12) 61 (8–12) 33 (12)
**D2 test of attention (Brickenkamp):**		
- GZ (pc) - % of errors (pc) - KL (pc)	187 (<0.1) 3.21 (50–75) 73 (4.5)	238 (0.5) 6.72 (25–50) 86 (8.1)

*Pc, percentiles; PANSS, Positive And Negative Symptoms Scale; AHRS, Auditory hallucinations rating scale; CGI, Global clinical impression; WAIS IV, Wechsler Adult Intelligence Scale (WAIS-IV); GZ, quantitative performance index; KL, concentration performance index*;

* *age corrected scores*.

There was a global improvement in a large part of neuropsychological measurements (episodic memory, executive and attentional functions) before and after stimulation (Table 1).

We found an improvement in the long-term episodic memory, assessed with the free/cued recall 16 items (Table 1) test. There was an increase of performance in the delayed free recall: after the stimulation, the score of the participant was in the normal range (19–20 percentile) vs. <1 percentile before stimulation. Likewise, there was a strong improvement in the first attempt of the free recall after stimulation: the score of the participant was in the normal range [11–12 percentile) after stimulation vs. 1–2 percentile before stimulation. Two different lists of words were proposed to the participant before and after stimulation in order to avoid a test-retest effect (21).

In two tests measuring short term and working memory (digit span and spatial memory), there was an improvement in the performance of the participant in both the direct and indirect order, suggesting an effect of stimulation in both the short term and working memory.

We found an increase in the Stroop test performance. In particular, the reading performance improved from 4 to 5 percentile (pathological range) before stimulation to 8–12 percentile (normal range) after stimulation. In addition there was an improvement in the interference condition after stimulation (12 percentile) as compared to before stimulation (5–8 percentile).

Last, we measured the selective attention with the D2 test of attention (Brickenkamp). Again, there was an increase in both the quantitative performance index (GZ) and the concentration performance index (KL). In the KL index, the subject scored in the normal range after cerebellar stimulation vs. in the pathological range (<0.5 percentile) before stimulation.

**Figure 2:**
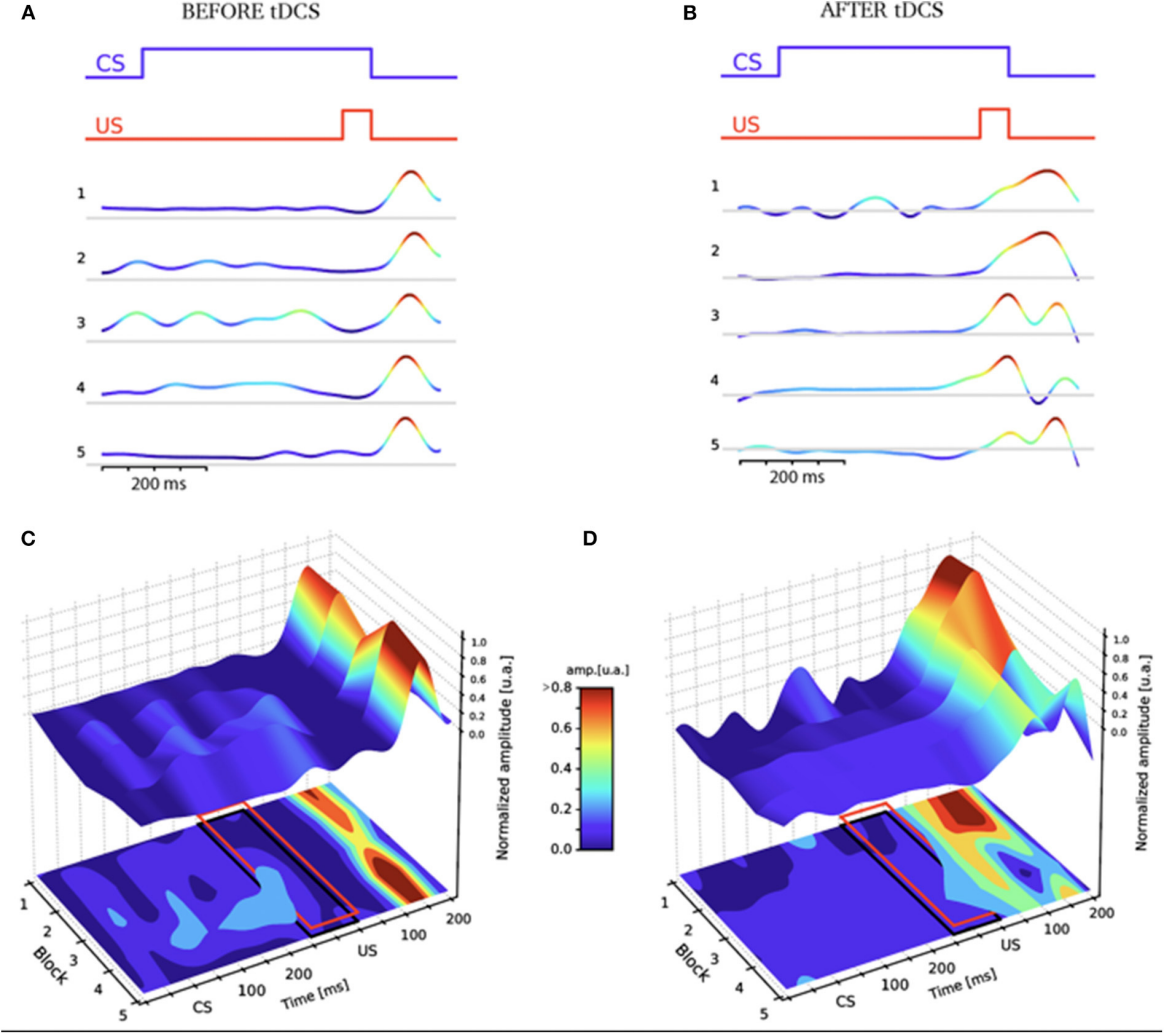
EBC sessions before **(A,C)** and after **(B,D)** tDCS in the schizophrenic patient. The color code corresponds to the normalized response amplitude of the IR-reflected signal. The EBC protocol is depicted at the top of **(A,B)**. **(A,B)** Each block represents the average signal over the 9 CS-US trials, for experiments respectively before **(A)** and after **(B)** tDCS. **(C,D)** Plots of the IR-reflected signal for each block before **(C)** and after **(D)** tDCS. Red rectangles delimit the area where CRs were detected in Figure 3 left. Notice that the unconditioned response (UR) peak amplitude and latency decreases block after block after tDCS **(D)**, while before the tDCS the UR peak amplitude remains constant among all the blocks, and the latency shows no trend **(C)**.

**Figure 3:**
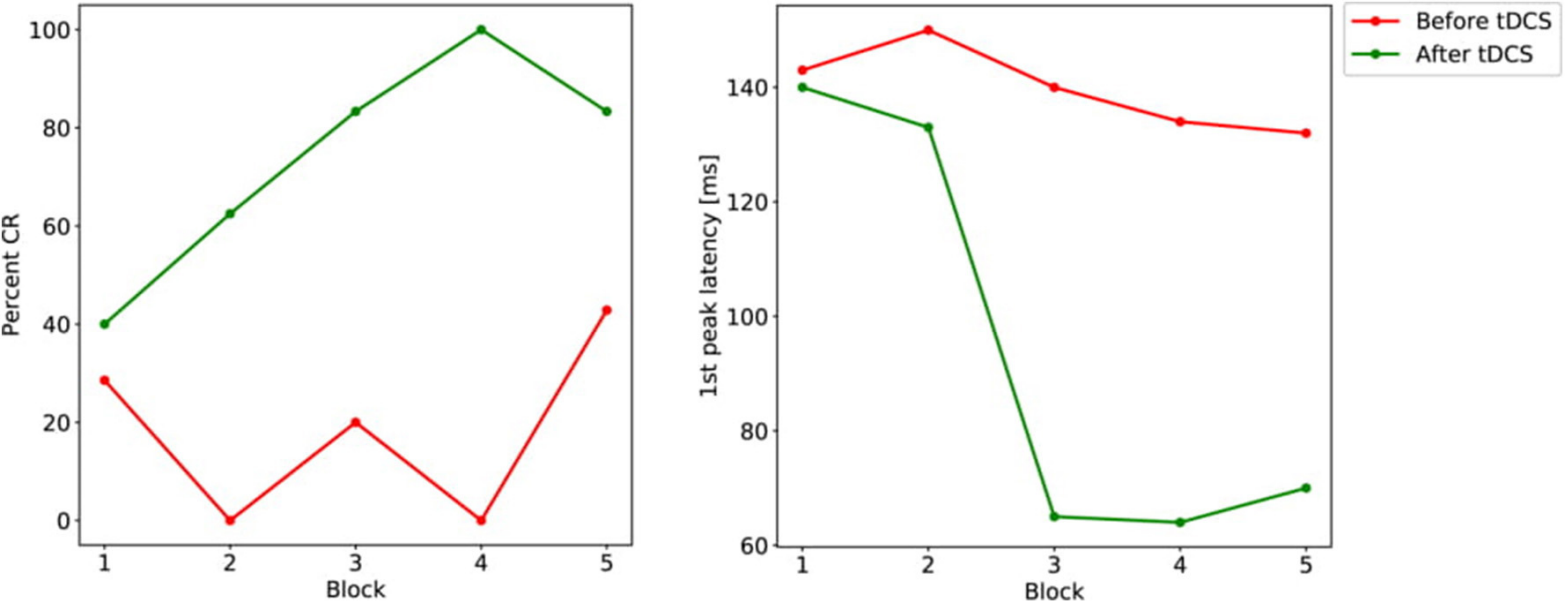
Percentage of CRs and latency of the first peak progression from block to block before (red) and after (green) tDCS. **Left panel:** percentage of CRs from each block measured in the red rectangle indicated on Figures 2C,D. **Right panel:** First peak latency calculated from the data averaged in blocks in Figures 2A,C. Time zero for latency calculation corresponds to the onset of the US.

Results from the EBC assessment before and after stimulation are reported in Figures 2, 3. Before tDCS, the averaged block response amplitudes of the URs remained unchanged over the pre-tDCS session (Figure 2A), very few CRs were detected and peak latencies of averaged block signals remained constant during the overall session (Figure 3). Thus, 45 CS-US pairings were insufficient to induce EBC, at the end of this session only 43% of the trials displayed CRs (Figure 3 left). This result is in agreement with previous EBC evaluation in patients with schizophrenia (22). After tDCS, the patient was rapidly conditioned and reached a final value of 83% of CRs (Figure 3 left), as expected for a normal EBC session. Accordingly after tDCS, the averaged amplitude of the URs decreased from block to block (Figure 2D) while it was stable before tDCS, this progression of EBC can also be observed by monitoring the first peak latency of the responses from block to block. Before tDCS it remained stable but rapidly decreased over the blocks after tDCS (Figure 3 right). Those features indicate the shift of the eyeblink timing toward the CS, which corresponds to a progressive change from *reflexive* toward *predictive* behavior. Thus, before tDCS the patient could not be conditioned over the EBC session while after cerebellar tDCS he displayed progressive conditioning from block to block.

## Discussion

We describe the case of a 50 years old patient with schizophrenia whom underwent a non-invasive cerebellar stimulation protocol. Data from clinical and psychometric evaluations including long term verbal memory, executive, and attention functions were collected before and after the stimulation, as well as data from a cerebellar-dependent eyeblink conditioning protocol.

Although we did not report changes in the positive or negative symptoms of schizophrenia before and after stimulation, there was a global improvement in psychometric measurements after stimulation. We also found an improvement in the performance in selected attention, long/short term memory, working memory, and response inhibition; cognitive domains known to be altered in patients with schizophrenia.

There was a clear improvement of EBC after stimulation. In the absence of data on healthy subjects in our conditions, we can not exclude any retest effect (“saving”) in this improvement (23). This is however unlikely given that in our data: (1) no clear cut EBC could be observed at the end of the pre-tDCS session, and (2) conditioning gradually appeared during the post-tDCS EBC session, starting from an absence of predictive response (and thus showing no saving, Figure 3). In addition, and contrary to control subjects, patients with schizophrenia have been shown not to improve their performance during consecutive EBC sessions (24). Those lines of evidence therefore support the view that the improvement of EBC after tDCS was due to the stimulation itself and not to any retention of the first EBC session. Based on the abundant literature in both humans and animals (10, 25–27), our data indicates that the cerebellar function of the patient was basally impaired as previously described in schizophrenia (28). Our EBC assessment is consistent with several studies that reported an effect of non-invasive cerebellar stimulation on associative learning measured with EBC (15, 29). More importantly, it points out cerebellar tDCS as a powerful tool to significantly improve cerebellar function in schizophrenia.

EBC has proven to be a relevant method to investigate cerebellar dysfunction in neuropsychiatric disorders. Disentangling motivational aspects from cognitive deficits can be challenging in patients with schizophrenia. This is however important since the deficits in motivation commonly present in patients with schizophrenia can bias classic neuropsychological tests. EBC does not require active participation of the subject. In our paradigm, the patient was watching a silent movie during the experiment; in newborns, EBC can even be performed during sleep (30, 31). Thus, the outcome of EBC are unlikely to be related to motivational deficits in patients with schizophrenia.

Gupta et al. (5) found in a double-blind crossover study an effect of cerebellar tDCS on procedural learning in a population of non-clinical psychosis (NCP) population. The authors reported greater rate of motor learning in NCP population after active stimulation. We used a different stimulation protocol with the cathode electrode (return electrode) placed on the right arm, whereas Gupta el al. placed the electrode on the midline of the scalp. Although there is no consensus on the placement of the return electrode (14), we chose this location based on a modelisation study (18) to target the posterior region of the cerebellum.

Brady et al. (32) reported in a population of patients with schizophrenia, an improvement of negative symptoms after transcranial magnetic stimulation (TMS) related to dorso-lateral prefrontal cortex-to-cerebellum connectivity. However, the authors did not investigate the effect of cerebellar stimulation on cognitive symptoms.

In healthy subjects, there is evidence that non-invasive cerebellar stimulation can modulate working memory, motor control, learning, and emotional processing (14). These results are in line with our case report where non-invasive cerebellar stimulation had an effect on verbal memory, executive, and attention function.

The participant did not report any significant side effects after 2 sessions of stimulation during 5 days, which is in line with previous studies showing the feasibility and good tolerance profile of cerebellar tDCS (14).

This case report supports several strengths. To the best of our knowledge, this case is the first to report the effect of tDCS on cognition (including associative learning measured with EBC) in schizophrenia. We carefully selected psychological measurements with no test/retest effects, which suggests that the cognitive improvement is related to the stimulation. In addition, there was no significant change in the positive and negative symptoms, suggesting again that the change in cognition are not related to a change in the symptoms of schizophrenia. Our work suggests that eyeblink conditioning can be used to assess the effect of cerebellar stimulation.

Several limits should be considered before interpreting our results. Because we only investigated the effect of stimulation in a single patient, our study remains purely qualitative. The posterior part of the cerebellum is connected to multiple regions in the associative cortex and it is difficult to target a specific domain of cognition with cerebellar brain stimulation. We were not able to measure other cognitive domains, such as social cognition that could also be modulated by cerebellar stimulation (33). However, our goal was to propose an original cognitive evaluation by combining a classic neuropsychological assessment and EBC.

In conclusion, this case report suggests that cerebellar tDCS stimulation can have an impact on cognitive impairments in patients with schizophrenia. We suggest that eyeblink conditioning, known as a relevant method to investigate cerebellar dysfunction in neuropsychiatric disorders, could be used to assess the impact of stimulation on the cerebellum in patients with schizophrenia. Further clinical trials are required to address the potential therapeutic potential of tDCS in schizophrenia.

## Data Availability Statement

The datasets generated for this study are available on request to the corresponding author.

## Ethics Statement

The studies involving human participants were reviewed and approved by Hôpital de Ville Evrard, Unité de Recherche Clinique. The patients/participants provided their written informed consent to participate in this study. Written informed consent was obtained from the participant for the publication of this case report.

## Author Contributions

CLa wrote the first draft of the manuscript. CLe setup and supervised the EBC experiments, contributed to the writing of the manuscript, and performed the EBC tests. AS-P wrote the Python routine, analyzed the EBC data, and contributed to the manuscript. FD participated in the selection of the neuropsychological tests and evaluated the patient. CF participated in the interpretation of neuropsychological assessment and to the writing of the manuscript. NB participated in the elaboration of the protocol, the clinical assessment, and the writing of the manuscript. DJ participated in the elaboration of the protocols and the writing of the manuscript.

### Conflict of Interest

The authors declare that the research was conducted in the absence of any commercial or financial relationships that could be construed as a potential conflict of interest.
